# Clinical factors associated with bloodstream infection at the emergency department

**DOI:** 10.1186/s12873-021-00426-2

**Published:** 2021-03-12

**Authors:** Pariwat Phungoen, Nunchalit Lerdprawat, Kittisak Sawanyawisuth, Verajit Chotmongkol, Kamonwon Ienghong, Sumana Sumritrin, Korakot Apiratwarakul

**Affiliations:** 1 Department of Emergency Medicine, Faculty of Medicine, Khon Kaen University, 123/2000 Mitraparp Rd, Muang, Khon Kaen, 40002 Thailand; 2 Department of Medicine, Faculty of Medicine, Khon Kaen University, Khon Kaen, 40002 Thailand; 3 Accidental and Emergency Unit, Division of Nursing, Srinagarind Hospital, Faculty of Medicine, Khon Kaen University, Khon Kaen, 40002 Thailand

**Keywords:** Bloodstream infection, Bacteremia, Blood cultures, Rapid diagnosis, Emergency department

## Abstract

**Background:**

Bloodstream infection (BSI) is a common urgent condition at the emergency department (ED). However, current guidelines for diagnosis do not specify the juncture at which blood cultures should be taken. The decision whether or not to obtain hemoculture is based solely upon clinical judgment and potential outcomes of inappropriately ordered cultures. This study aimed to find clinical factors present on ED arrival that are predictive of bloodstream infection.

**Methods:**

This study was conducted retrospectively at the ED of *a single tertiary* care *hospital* in Thailand. We included adult patients with suspected infection based on blood culture who were treated with intravenous antibiotics during their ED visit. Independent positive predictors for positive blood culture were calculated by logistic regression analysis.

**Results:**

A total of 169,578 patients visited the ED during the study period, 12,556 (7.40%) of whom were suspected of infection. Of those, 8177 met the study criteria and were categorized according to blood culture results (741 positive; 9.06%). Six clinical factors, including age over 55 years, moderate to severe CKD, solid organ tumor, liver disease, history of chills, and body temperature of over 38.3 °C, were associated with positive blood culture.

**Conclusions:**

Clinical factors at ED arrival can be used as predictors of bloodstream infection.

## Background

Bloodstream infection (BSI) is a common urgent condition at the emergency department (ED) [1, 2]. In 2010, the annual incidence of bloodstream infection increased to 38.1 persons per 100,000, and the mortality rate was as high as 50% [3]. Early diagnosis and appropriate antimicrobial therapy are a key to improving patient outcomes [4], particularly among individuals displaying either septic shock or sepsis [5, 6].

Current guidelines recommend obtaining hemoculture in patients suspected of sepsis in order to diagnose BSI [5, 7], as positive blood culture is an important factor in determining the appropriate antibiotic treatment [5, 8]. However, the guidelines do not specify when blood cultures ought to be procured. Furthermore, the decision as to whether to take hemoculture is based solely upon clinical judgment, which could result in unnecessary cultures [8–10]. There are several predictors of bloodstream infection at the ED such as blood pressure less than 60 mmHg, procalcitonin levels over 2 μg/L, and C-reactive protein> 10 mg/dL [11]. Shapiro et al. developed a clinical score for bloodstream infection at the ED with a decent validation of 83% [12]. However, obtaining this score may require laboratory results, which could result in delayed sepsis management [6]. Hence, this study examined only clinical factors present on ED arrival to determine which, if any, were predictive of bloodstream infection.

## Methods

### Study design and ethical approval

This study was conducted retrospectively as part of an ED infection project at Khon Kaen University’s Srinagarind Hospital, a tertiary care hospital with approximately 60,000 annual ED visits. Inclusion criteria were age > 18 years, suspicion of infection based on blood culture collection, and initiation of intravenous antibiotics during the ED visit. Cases with cardiac arrest or trauma, those referred from other hospitals, those who had previously received antibiotics, and those missing clinical data were excluded. The study period took place between January 1st, 2016 and December 31st, 2018. The study protocol was approved by the Khon Kaen University Ethics Committee in Human Research (HE631115).

### Source of data and microbiological methods

Blood cultures at the ED each consist of two aerobic bottles. Those with a pathogen similar to at least one sample with clinical relevance were considered positive for bloodstream infection. Pathogens (e.g., coagulase-negative Staphylococci, Corynebacterium spp., Propionibacterium spp., Viridans group streptococci, Micrococcus spp., and Bacillus spp.) were considered as such if they were isolated from a patient twice or more consecutively with clinical relevance [7, 13, 14]. Clinical data of eligible patients were retrieved from the computerized hospital database and chart records. Data were subsequently categorized as comorbid conditions, ED arrival parameters, and parameters beyond the initial hour following presentation at the ED. Comorbid conditions were defined according to the Charlson Comorbidity Index (CCI) [15]. ED arrival parameters included history of fever or chills, vital signs, and sepsis scores including Systemic Inflammatory Response Syndrome (SIRS) score, quick Sepsis-related Organ Failure Assessment (qSOFA), and National Early Warning Score (NEWS). Parameters beyond the initial hour post ED arrival included white blood cell count and lactate levels.

### Statistical analysis

Eligible patients were categorized into two groups based on whether their blood culture results were positive or negative. Descriptive statistics were used to compare differences in studied variables between the two groups. Factors associated with positive blood culture were calculated via logistic regression analysis. Univariate and multivariate logistic regression were applied to calculate the unadjusted/adjusted odds ratios (95% confidence interval) of each factor. All statistical analyses were performed using STATA version 10.1 (College Station, Texas, USA).

## Results

### Patient characteristic and microbiology data

**Fig. 1:**
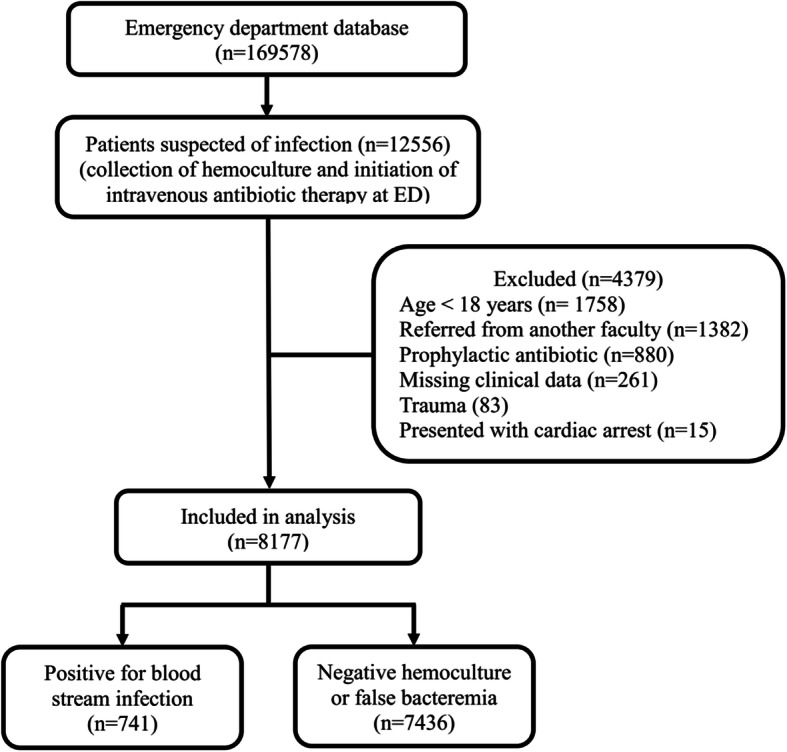
Study flow of patients with suspected infection presenting at the emergency department and blood culture results

A total of 169578 patients visited the ED during the study period, of which 12556 (7.40%) were suspected of infection according to the hospital database. After exclusion, 8177 individuals met the study criteria and were categorized according to blood culture results as either positive for bloodstream infection (741 patients; 9.06%) or negative/non-pathogen bacteremia (7436 patients; 90.94%), as shown in Fig. 1. Almost all variables studied differed significantly between groups (Table 1), with the exception of AIDS prevalence (2.16% in the positive group and 1.44% in the negative group; p 0.125). The most common Gram-negative and positive pathogens were *Escherichia coli* (274 patients; 36.98%) and Streptococcus (76 patients; 10.26%), respectively.

**Table 1:** Baseline characteristics of patients with suspected infection presenting at the emergency department categorized by blood culture results

	ALL patients (***n*** = 8177) ***n*** (%)	Positive blood culture (***n*** = 741) ***n*** (%)	Negative blood culture (***n*** = 7436) ***n*** (%)	***p***-value
**Demographics**				
Age, yrs. –median (range)	62 (18–100)	62 (18–100)	64 (18–100)	< 0.001
Male	4275 (52.28)	415 (56.01)	3860 (51.90)	0.003
CCI –median (range)	3 (0–13)	4 (0–13)	3 (0–13)	< 0.001
**Comorbidity**				
Age > 55 years	5231 (63.97)	537 (72.47)	4694 (63.13)	< 0.001
Hypertension	2149 (26.28)	235 (31.71)	1914 (25.74)	< 0.001
Solid organ malignancy	1878 (22.97)	231 (31.17)	1647 (22.15)	< 0.001
Diabetes mellitus	1729 (21.14)	194 (26.18)	1535 (20.64)	< 0.001
Liver disease	1190 (14.55)	191 (25.78)	999 (13.43)	< 0.001
Moderate to severe CKD	639 (7.81)	86 (11.61)	553 (7.44)	< 0.001
AIDS	123 (1.50)	16 (2.16)	107 (1.44)	0.125
**History of chills**	515 (6.30)	101 (13.63)	414 (5.57)	< 0.001
**Clinical presentation at triage zone**				
Respiratory rate > 22/min	5369 (65.66)	573 (77.33)	4796 (64.50)	< 0.001
Temperature > 38.3 °C	2658 (32.51)	349 (47.10)	2309 (31.05)	< 0.001
Heart rate > 120/min	921 (11.26)	105 (14.17)	816 (10.97)	< 0.001
Hypotension (SBP < 90 or MAP < 65 mmHg)	611 (7.47)	96 (12.96)	515 (6.93)	< 0.001
**Met Sepsis criteria**				
SIRS ≥2	6149 (75.20)	651 (87.85)	5498 (93.94)	< 0.001
qSOFA ≥2	1230 (15.04)	140 (18.89)	1060 (14.25)	< 0.001
NEWS ≥7	2917 (35.67)	259 (34.95)	1759 (23.66)	< 0.001
**Lactate values**	(*n* = 4694)	(*n* = 575)	(*n* = 4119)	
First lactate, mmol/L-median	1.88 (0.01–28.33)	2.50 (0.01–18.71)	1.80 (0.01–28.33)	< 0.001
First lactate > 2 mmol/L	2193 (46.72)	383 (51.69)	1810 (23.34)	< 0.001

*CKD* chronic kidney disease, *SBP* systolic blood pressure, *MAP* mean arterial pressure, *SIRS* Systemic Inflammatory Response Syndrome, *qSOFA* quick Sepsis-related Organ Failure Assessment, *NEWS* National Early Warning Score

### Clinical factors predictive of bloodstream infection

There were four significant comorbid conditions, two factors at ED arrival, and three factors beyond the first hour (Table 2). The six significant predictors for positive blood culture on ED arrival were age over 55 years, moderate to severe CKD, solid organ tumor, liver disease, history of chills, and body temperature over 38.3 °C. Liver disease had the highest adjusted odds ratio at 2.04 (95% CI of 1.59, 2.61). The adjusted odds ratio of independent factors ranged from 1.33 to 2.04 (Table 2). Beyond the first hour after ED arrival, lactate level and white blood cell count were both significant factors, with adjusted odds ratios ranging from 1.10 to 2.48.

**Table 2:** Factors associated with positive blood culture in patients suspected of infection presenting at the emergency department

Factors	Unadjusted Odds Ratio (95% CI)	^a^Adjusted Odds Ratio (95% CI)	*p*-value
**Comorbid conditions**			
Age > 55	1.54 (1.30–1.81)	1.33 (1.04–1.72)	0.02
Sex	0.84 (0.73–0.99)	0.94 (78.1–1.13)	0.52
Emergency severity index level	0.62 (0.55–0.70)	0.87 (0.71–1.13)	0.18
CCI	1.13 (1.09–1.06)	0.98 (0.92–1.04)	0.46
Liver disease	2.24 (1.87–2.67)	2.04 (1.59–2.61)	< 0.01
Diabetes mellitus	1.36 (1.15–1.62)	1.08 (0.89–1.30)	0.45
Moderate to severe CKD	1.63 (1.28–2.08)	1.68 (1.22–2.32)	0.01
Solid organ tumor	1.59 (1.35–1.87)	1.40 (1.09–1.80)	0.01
Hypertension	1.34 (1.13–1.57)	1.14 (0.92–1.41)	0.24
**On arrival parameter**			
History of Chills	2.67 (2.12–3.38)	1.94 (1.43–2.62)	< 0.01
Temperature > 38.3 °C	1.40 (1.32–1.50)	1.77 (1.39–2.25)	< 0.01
Heart rate > 120 /min	1.01 (1.00–1.01)	0.96 (0.73–1.26)	0.76
SBP < 90 or MAP < 65	2.01 (1.59–2.54)	1.22 (0.86–1.71)	0.26
Respiratory rate > 22/min	1.03 (1.02–1.04)	0.89 (0.71–1.26)	0.32
SIRS criteria ≥2	2.5 (2.03–3.20)	1.21 (0.94–1.53)	0.26
qSOFA criteria ≥2	1.79 (1.49–2.15)	1.20 (0.94–1.53)	0.15
NEWS ≥7	1.73 (1.47–2.04)	0.93 (0.73–1.18)	0.53
**Beyond first hour parameter**			
^b^Lactate level	1.13 (1.10–1.16)	1.10 (1.07–1.14)	< 0.01
WBC > 11,000 /microliter	1.31 (1.12–1.52)	1.28 (1.03–1.59)	0.03
WBC < 3000 /microliter	2.30 (1.70–3.13)	2.48 (1.68–3.66)	< 0.01

*CCI* Charlson Comorbidity Index, *CKD* chronic kidney disease, *SBP* systolic blood pressure, *MAP* mean arterial pressure, *SIRS* Systemic Inflammatory Response Syndrome; *qSOFA* quick Sepsis-related Organ Failure Assessment, *NEWS* National Early Warning Score, *WBC* white blood cell

^a^ adjusted by the studied factors shown in this table

^b^ initial lactate level in mmol/L.

## Discussion

The positive blood culture rate in this study (9.06%) was comparable with those in previous studies (up to 12.4%) [11, 16]. Subjecting low-risk patients to unnecessary blood culture may yield false positives and increase healthcare costs [12]. As previously reported [12, 17–19], fever and older age are independently associated with positive blood culture at the ED. Although body temperature over 38.3 degrees celsius is one criterion included in the SIRS score [5], only 47.10% of patients in this group had positive cultures (Table 1). The proportion of patients in the positive group with respiratory rate over 22 times/min was higher than that of those with high body temperature (77.33% vs 47.10%). However, this difference was not significant after adjustment for other factors (Table 2). This implies that body temperature alone may not be an adequate indicator of positive blood culture and that it should, instead, be considered in combination with the other five independent factors.

History of chills, which is an indicator of pyrogenic cytokines, is another predictor of positive blood culture. Previous studies by Tokuda et al. and Holmqvist et al. showed history of chills to be associated with positive blood culture, regardless of severity (mild to shaking) [20, 21]. However, these studies had smaller populations and adjusted for fewer other variables than did our study. The former adjusted for only age and body temperature (*n* = 526), while the latter adjusted for age, sex, vomiting, and antibiotic use (*n* = 479).

This study’s findings regarding co-morbid diseases and laboratory tests differed from those of some previous studies [17, 18]. One study, for example, found that the prevalence of liver cirrhosis, chronic kidney disease, and malignancy did not differ significantly between those with and those without positive blood culture [17], whereas our study found a correlation between these co-morbid diseases and blood culture positivity. These differences may have been due to our larger study population or the fact that we used clinical factors (with no laboratory results) to predict positive blood cultures in order to allow for more rapid assessment of risk. However, there have been other studies that have reported findings similar to ours [19, 22–24]. For example, one previous study found that cirrhotic patients had a higher incidence of bloodstream infection than non-cirrhotic patients [24].

### Strengths and limitations

In this study, we enrolled a large population to determine predictors for positive blood culture in ED patients with suspicion of infection. However, there were some limitations to this study. Although we employed a large sample size, clinical data were missing in some cases due to the retrospective study design. Such cases were excluded (261 patients). Second, blood cultures were performed based on the judgement of the attending physicians at a single ED. Further studies are thus required to validate and confirm the results of this study. Finally, the results may not be universal for other setting such as community hospitals [25, 26].

## Conclusions

Six clinical factors, including age over 55 years, moderate to severe CKD, solid organ tumor, liver disease, history of chills, and body temperature of over 38.3 °C were associated with blood culture positivity. Consideration of these clinical factors may allow for more rapid assessment of positive blood culture risk in ED patients suspected of infection.

## Data Availability

The datasets used and/or analyzed during the current study are available from the corresponding author on reasonable request.

## Abbreviations

AUROC: Associated area under the ROC
BSI: Bloodstream infection (BSI)
CCI: Charlson Comorbidity Index
CKD: Chronic kidney disease
ED: Emergency department
+LR: Positive likelihood ratio
-LR: Negative likelihood ratio
MAP: Mean arterial pressure
NEWS: National Early Warning Score
qBSI score: Quick Bloodstream Infection score
ROC curve: Receiver operating characteristic curves
SIRS: Systemic Inflammatory Response Syndrome (SIRS)
SBP: Systolic blood pressure
